# Case report: Pain in anti-DPPX encephalitis

**DOI:** 10.3389/fneur.2022.1091688

**Published:** 2022-12-14

**Authors:** Tale L. Bjerknes, Ole Martin Steihaug, Mette Haugen, Ina Elen Hjelland, Christian Alexander Vedeler

**Affiliations:** ^1^Neuro-SysMed, Department of Neurology, Haukeland University Hospital, Bergen, Norway; ^2^Emergency Clinic, Haukeland University Hospital, Bergen, Norway; ^3^Department of Neurophysiology, Haukeland University Hospital, Bergen, Norway; ^4^Department of Clinical Medicine, University of Bergen, Bergen, Norway

**Keywords:** anti-dipeptidyl-peptidase-like protein 6 (DPPX), autoimmune encephalitis, weight loss, extremity pain, rituximab

## Abstract

Encephalitis due to antibodies targeting dipeptidyl-peptidase-like protein 6 (DPPX), a potassium channel subunit, is rare. The illness is typically characterized by a triad of weight loss, CNS hyperexcitability and cognitive symptoms, but recent reports suggest that the clinical picture may be more heterogeneous. Here, we describe the case of a 63-year-old female who was admitted to the hospital with severe extremity pain, which had been preceded by diarrhea and weight loss. She later developed cognitive changes, and her general condition rapidly deteriorated. Extensive workup did not reveal gastrointestinal illness or underlying malignancies. MRI of the brain was normal. Analyses of blood and cerebrospinal fluid showed normal cell counts but high titres of DPPX antibodies in blood and cerebrospinal fluid. The patient was treated with intravenous methylprednisolone followed by rituximab. At 1-year follow-up, she was without pain and had completely recovered. In this case, DPPX-associated autoimmune encephalitis was dominated by severe extremity pain, illustrating that sensory symptoms may be one of the main complaints in these patients. It is important for clinicians to be aware of the heterogeneous clinical picture in this serious condition, since correct diagnosis and treatment with immunosuppressants are associated with favorable prognosis.

## Introduction

Encephalitis associated with anti-dipeptidyl-peptidase-like protein 6 (DPPX) is a rare condition associated with weight loss, gastrointestinal symptoms, and neurological symptoms (1, 2). Major features include cognitive changes and central nervous system (CNS) hyperexcitability, as well as cerebellar symptoms and sleep disturbances (2). Symptoms are thought to arise due to antibodies that bind to the cell-surface antigen DPPX, which is a subunit of the Kv4.2. potassium channel (3). DPPX is expressed in many brain areas, including the hippocampus and cerebellum, as well as in the myenteric plexus (1). Reduced expression of DPPX and thus decreased Kv4.2 potassium channel activity in the gastrointestinal tract accompanied by neuronal hyperexcitability may be the cause of gastrointestinal symptoms and weight loss in these patients (2). In this paper, we describe a case of DPPX antibody-associated autoimmune encephalitis in which severe extremity pain was the major complaint; the patient also suffered from diarrhea, weight loss, and cognitive changes.

## Case presentation

**Figure 1:**
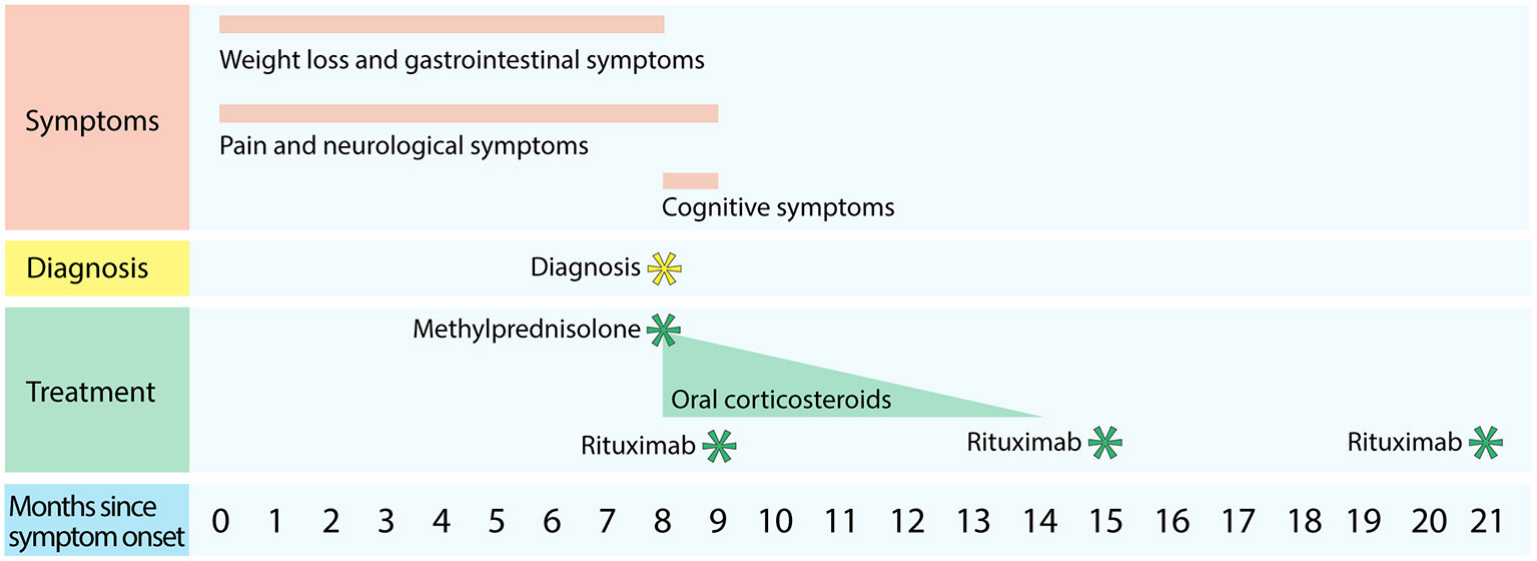
Time course of symptoms, diagnosis, and treatment.

A 63-year-old female was referred to Haukeland University Hospital after she developed painful, burning sensations and paresthesia, primarily in the extremities. A timeline of the symptoms and treatment are shown in Figure 1. The symptoms had progressed over 4 to 6 months prior to the hospitalization. In the same period, she had felt nauseous, had little appetite, and had developed diarrhea leading to a weight loss of 18 kg. The patient also complained of headache, dizziness, and insomnia. At night, she often walked on a cold floor to get some relief from her lower extremity pain. All her extremities felt heavy, and she complained of muscle ache. She also developed a red erythema on her chest and had slight general pruritus. Her earlier medical history included migraine, gout, gastroesophageal reflux, and cholecystectomy, however she was active and lived independently in her home.

The patient was extensively examined without findings of gastrointestinal illness. She had a normal gastroscopy and colonoscopy with normal biopsies and no signs of coeliac disease or a neuroendocrine tumor. In initial workup, blood tests were normal with only slightly elevated hemoglobin and ferritin levels. Tests were negative for antinuclear antibodies, and protein electrophoresis was normal. An infectious agent could not be identified. In addition, a broad endocrinology workup was normal. The patient was also screened for underlying malignancies. Total body CT scan showed multiple, partly cystic changes in the uterus, and further gynecological evaluation confirmed the presence of myomas with normal cervical cytology and endometrial biopsy samples. Whole body PET-CT scan and brain MRI were normal.

During the months after her first hospitalization, the patient's general condition worsened with progression of weight loss and increasing apathy. Throughout the course of the disease, she had persistent severe pain and allodynia as her major complaint, which restricted her daily life significantly. Clinical neurological examination at this stage was normal. Electromyography showed active denervation and nerve conduction study showed slight axonal and demyelinating motor and sensory changes in the lower extremity. Electro-encephalography showed frequent focal low-frequency activity on the left frontotemporal area, both arrhythmic and single waves. There were also short second-long sequences with diffuse low-frequent activity.

5 months after the start of her diagnostic evaluation, the patient was unable to take care of herself. She was underweight with a BMI of 18, spent most of her days confined to the bed, and was not able to walk without support. At this stage, she showed reduced facial expressions, a low mood, and significant apathy and fatigue. She also had memory deficits with problems recalling details of her medical history. She repeated the same sentences, had difficulties changing topics during conversation, and had difficulties concentrating. Her gait was unsteady with some truncal ataxia but without tendency to fall. She had slightly increased tone in the extremities but no symptoms of cerebral hyperexcitability. Upper and lower extremity pain continued to be a major complaint.

**Figure 2:**
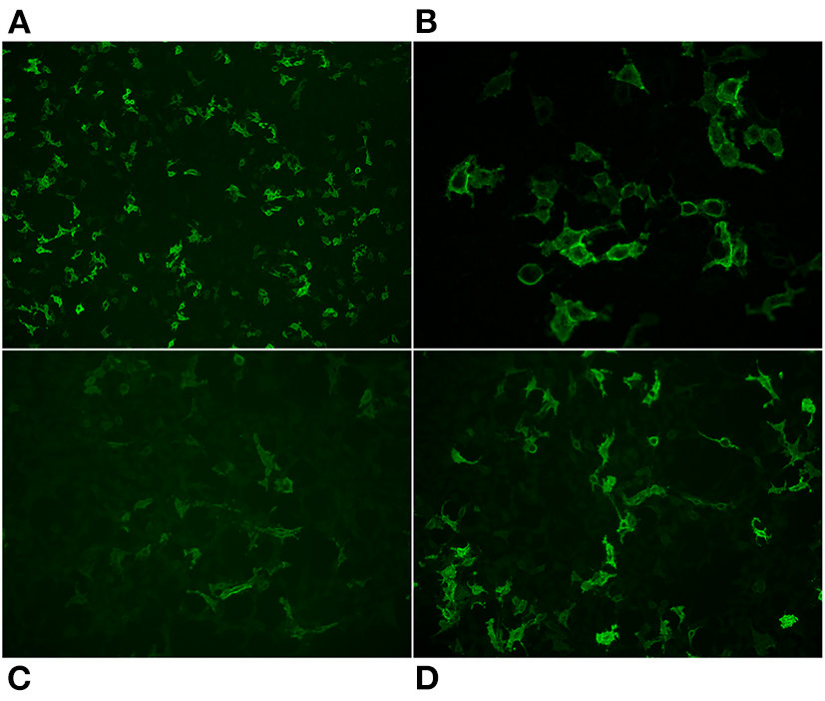
Representative images of the reaction of antibodies from the patients' serum with cells that express DPPX. Total IgG staining 10x **(A)** 40x **(B)**, IgG2 20x **(C)**, IgG4 20x **(D)**.

Routine screening of blood was normal. Analysis of cerebrospinal fluid showed normal cell count and moderately increased level of protein. High titres of anti-DPPX antibodies were found in serum (10^6^) and cerebrospinal fluid (10^3^) (Figures 2A,B). The IgG subclass distribution of anti-DPPX in both serum and cerebrospinal fluid was: IgG4>>IgG2>IgG1=IgG3 (Figures 2C,D). No other encephalitis or paraneoplastic antibodies were detected using the Euroimmune autoimmune encephalitis mosaic 6 cell-based assay or the Ravo PNS 14 line assay. Her condition was assessed as probable DPPX antibody-associated autoimmune encephalitis with severe weight loss, and she was started on high-dose intravenous corticosteroids (methylprednisolone 1 g) for 5 days with subsequent slow dose tapering of prednisolone over several months. Initial treatment with methylprednisolone improved the patient's condition. She was subsequently treated with an infusion of rituximab (1,000 mg) beginning 1 month after discharge and with two additional rituximab treatments (500 mg each) at 6-month intervals. She was discharged from the hospital into a rehabilitation institution. After slow and gradual improvement, she returned to her home after several months. Serum tested 7 months after the initial antibody screening showed a decrease in the anti-DPPX titer (10^4^). At a year after treatment initiation with corticosteroid, she had gained weight, completely recovered from the pain and other neurological complaints, and lived independently in her home performing all activities of daily living. Her neurophysiological changes were also normalized. She has since been without any immune therapy.

## Discussion

Autoimmune encephalitis refers to a group of conditions with varying symptomatology caused by autoantibodies toward cell-surface antigens or synaptic antigens such as neurotransmitter receptors or ion channels (4). Encephalitis caused by antibodies that bind to the Kv4.2 potassium channel subunit DPPX was described by Boronat in 2013; symptoms reported included weight loss and gastrointestinal symptoms, CNS hyperexcitability, cognitive changes, sleep disturbances, and cerebellar symptoms (1). DPPX is expressed in areas such as the hippocampus, cerebellum, and myenteric plexus (1).

To date, 65 patients with encephalitis caused by antibodies to DPPX have been described in the literature (1, 2, 5–23) (Table1 and Supplementary Table S1). The median age of these patients at diagnosis was 52 (range 13–80), and 66 % of the reported cases were male. The most commonly reported symptoms are cognitive, reported in 56 of 65 patients (86%). Also common were CNS hyperexcitability (49/65 patients; 75%) and weight loss and/or gastrointestinal symptoms (47 of 65 patients; 72%). A little less than half of the reported patients experienced cerebellar symptoms and sleep alterations, whereas dysautonomia and brain stem disorders were more seldom reported. Few papers have previously reported sensory symptoms, and only one has described pruritus as the main feature of this rare disease (2, 6, 18, 22). The case described here was a novel clinical presentation of DPPX antibody-associated encephalitis where altered pain perception was the primary symptom in addition to more common features such as weight loss and cognitive alterations. Our patient also complained of some pruritus, but to a much lesser degree than previously reported.

**Table 1:** Reported symptoms in 65 patients with DPPX antibody-associated encephalitis^*)^ ^**)^.

**Symptoms**	**Number of patients with symptom (%)**	**Female/male**
Gastrointestinal symptoms and/or weight loss	47 (72 %)	15/31^***)^
Cognitive dysfunction	56 (86 %)	18/37^***)^
Psychiatric symptoms	24 (37 %)	12/12
CNS hyperexcitability (myoclonus, tremor, hyperreflexia seizures)	49 (75 %)	13/35^***)^
Paresis	1 (2 %)	1/0
Cerebellar dysfunction	29 (45 %)	12/17
Brain stem disorders	16 (25 %)	5/11
Dysautonomia	19 (29 %)	5/11^****)^
Sleep disorders	26 (40 %)	11/15
Sensory symptoms (allodynia, pruritus)	5 (8%)	0/5

*) Based on the following studies 1, 2, 5–23.

**) Details given in Supplementary Table S1.

***) Gender not reported for one patient.

****) Gender not reported for three patients.

The Kv4.2. potassium channel is present in the dorsal horn neurons, and a previous study found that genetic elimination of the 4.2 potassium channel subunit in mice led to increased sensitivity to tactile and thermal stimuli (24). It has been shown in animal models that antibody-mediated or genetic ablation of DPPX may result in neuronal hyperexcitability (5). Of interest is that our patient had electrophysiological changes of active denervation that normalized during treatment. However, since the patient did not show neurophysiological changes expected based on her pain levels, it is thus possible that alterations in spinothalamic signals also contributed to her pain sensitivity and pruritus.

Most reported patients with anti-DPPX mediated autoimmune encephalitis respond to immunotherapy, some many months after onset of symptoms (7). The most commonly used first-line therapy is corticosteroids (2). Other first-line therapies include intravenous immunoglobulin therapy (IVIg) and plasma exchange, which can be given alone or in combination with corticosteroids. Treatment with corticosteroids may in some cases result in complete recovery but relapses are also common (2). Second-line therapy with B cell-targeted agents such as rituximab is often needed to prevent relapse. Rituximab is especially effective in treatment of IgG4-mediated illnesses, and the antibodies toward DPPX are predominately IgG1 and IgG4, but also IgG2 (2, 25). We found that the main subclasses of anti-DPPX was IgG4 and IgG2. Our patient recovered completely after initiation of second-line therapy with rituximab, and a year after her first admission, she had completely recovered from the extremity pain and other neurological symptoms. This case thus shows that pain may be a prominent feature of encephalitis associated with anti-DPPX antibodies, and our patient is an example of the large variation of symptomatology in this rare disorder. It is important for clinicians to be aware of the clinical heterogeneity of the disorder to avoid delay in diagnosis and initiation of effective treatment.

## Data availability statement

The original contributions presented in the study are included in the article/Supplementary material, further inquiries can be directed to the corresponding author.

## Ethics statement

Written informed consent was obtained from the individual(s) for the publication of any potentially identifiable images or data included in this article.

## Author contributions

TB and CV wrote the article with input from all authors. All authors contributed to the work-up, treatment of the patient, reviewed the manuscript, and approved the final version.
