# Supplementary material for: Case report: Pain in anti-DPPX encephalitis
Source: Front Neurol. 2022 Dec 14;13:1091688. doi: 10.3389/fneur.2022.1091688 (PMC9794743; doi:10.3389/fneur.2022.1091688)
Supplement: Supplementary file 1 [file Table_1.pdf]

Table S1. Details on reported symptoms in 65 patients with DPPX antibody-associated encephalitis

Signs and symptoms	Number of patients with symptom	References	Female/male
Gastrointestinal symptoms and/or weight loss	2	Boronat et al 2013	1/1
	2	Balint et al 2014	0/2
	15	Tobin et al 2014	5/10
	1	Piepgras et al 2015	0/1
	1	Stoeck et al 2015	1/0
	1	Stokin et al 2015	0/1
	1	Dohety et al 2017	0/1
	1	Valero-Lopez et al 2017	1/0
	9	Hara et al 2017	2/7
	1	Zhou et al 2020	0/1
	1	Deuel et al 2020	0/1
	1	Wjintes et al 2020	0/1
	1	Ye et al 2021	0/1
	2	Mbonde et al 2021	1/1
	1	Tsai et al 2021	0/1
	1	Swayne 2021	N/A
	5	Xiao et al 2022	4/1
	1	Legendre et al 2022	0/1
	**Total 47**		**Total 15/31**
Cognitive dysfunction	3	Boronat et al 2013	2/1
	2	Balint et al 2014	0/2
	16	Tobin et al 2014	5/11
	1	Piepgras et al 2015	0/1
	1	Stoeck et al 2015	1/0
	1	Stokin et al 2015	0/1
	1	Dohety et al 2017	0/1
	1	Valero-Lopez et al 2017	1/0
	9	Hara et al 2017	2/7
	1	Bien et al 2020	1/0
	1	Zhou et al 2020	0/1
	1	Deuel et al 2020	0/1
	1	Wjintes et al 2020	0/1
	1	Ye et al 2020	0/1
	2	Mbonde et al 2021	1/1
	1	Tsai et al 2021	0/1
	1	Swayne 2021	N/A
	7	Xiao et al 2022	5/2
	1	Legendre et al 2022	0/1
	4	Miao et al 2022	0/4
	**Total 56**		**Total 18/37**
Psychiatric symptoms	3	Boronat et al 2013	2/1
	5	Tobin et al 2014	4/1
	1	Stoeck et al 2015	1/0
	1	Stokin et al 2015	0/1
	1	Valero-Lopez et al 2017	1/0
	6	Hara et al 2017	1/5
	1	Zhou et al 2020	0/1
	1	Bien et al 2020	1/0
	1	Deuel et al 2020	1/0
	1	Mbonde et al 2021	1/0
	1	Tsai et al 2021	0/1
	2	Xiao et al 2022	0/2
	**Total 24**		**Total 12/12**

CNS hyperexcitability (myoclonus, tremor, hyperreflexia, seizures)	3<br>3<br>11<br>1<br>1<br>1<br>1<br>1<br>8<br>1<br>1<br>1<br>1<br>2<br>1<br>1<br>7<br>4<br>**Total 49**	Boronat et al 2013<br>Balint et al 2014<br>Tobin et al 2014<br>Piepgras et al 2015<br>Stoeck et al 2015<br>Stokin et al 2015<br>Dohety et al 2017<br>Valero-Lopez et al 2017<br>Hara et al 2017<br>Bien et al 2020<br>Deuel et al 2020<br>Wjintes et al 2020<br>Ye et al 2021<br>Mbonde et al 2021<br>Tsai et al 2021<br>Swayne et al 2021<br>Xiao et al 2022<br>Miao et al 2022	2/1<br>0/3<br>1/10<br>0/1<br>1/0<br>0/1<br>0/1<br>1/0<br>2/6<br>1/0<br>0/1<br>0/1<br>0/1<br>1/1<br>0/1<br>N/A<br>4/3<br>0/4<br>**Total 13/35**
Paresis	1	Tobin et al 2014	1/0
Cerebellar dysfunction	3<br>9<br>1<br>1<br>1<br>1<br>4<br>1<br>1<br>1<br>2<br>2<br>1<br>1<br>**Total 29**	Balint et al 2014<br>Tobin et al 2014<br>Piepgras et al 2015<br>Stoeck et al 2015<br>Dohety et al 2017<br>Valero-Lopez et al 2017<br>Hara et al 2017<br>Deuel et al 2020<br>Wjintes et al 2020<br>Ye et al 2021<br>Mbonde et al 2021<br>Xiao et al 2022<br>Miao et al 2022<br>Lin et al 2022	0/3<br>4/5<br>0/1<br>1/0<br>0/1<br>1/0<br>3/1<br>0/1<br>0/1<br>0/1<br>1/1<br>2/0<br>0/1<br>0/1<br>**Total 12/17**
Brain stem disorders	15<br>1<br>**Total 16**	Tobin et al 2014<br>Miao et al 2022	5/10<br>0/1<br>**Total 5/11**
Dysautonomia	2<br>10<br>2<br>1<br>1<br>3<br>**Total 19**	Balint et al 2014<br>Tobin et al 2014<br>Hara et al 2017<br>Wjintes et al 2020<br>Mbonde et al 2021<br>Xiao et al 2022	0/2<br>3/7<br>1/1<br>0/1<br>1/0<br>N/A<br>**Total 5/11**
Sleep disorders	1<br>9<br>1<br>1<br>1<br>2<br>1<br>1	Boronat et al 2013<br>Tobin et al 2014<br>Piepgras et al 2015<br>Dohety et al 2017<br>Valero-Lopez et al 2017<br>Hara et al 2017<br>Zhou et al 2020<br>Deuel et al 2020	1/0<br>3/6<br>0/1<br>0/1<br>1/0<br>2/0<br>0/1<br>0/1

	1<br>2<br>5<br>1<br>**Total 26**	Ye et al 2021<br>Mbonde et al 2021<br>Xiao et al 2022<br>Miao et al 2022	0/1<br>1/1<br>3/2<br>0/1<br>**Total 11/15**
Sensory (allodynia, pruritus)	2<br>1<br>1<br>1<br>**Total 5**	Balint et al 2014<br>Hara et al 2017<br>Wjintes et al 2020<br>Legendre et al 2022	0/2<br>0/1<br>0/1<br>0/1<br>**Total 0/5**
